# Dose–Response Efficacy and Mechanisms of Orally Administered *Bifidobacterium breve* CCFM683 on IMQ-Induced Psoriasis in Mice

**DOI:** 10.3390/nu15081952

**Published:** 2023-04-18

**Authors:** Xinqi Chen, Yang Chen, Catherine Stanton, Reynolds Paul Ross, Jianxin Zhao, Wei Chen, Bo Yang

**Affiliations:** 1 State Key Laboratory of Food Science and Technology, Jiangnan University, Wuxi 214126, China; 2 School of Food Science and Technology, Jiangnan University, Wuxi 214126, China; 3 International Joint Research Center for Probiotics & Gut Health, Jiangnan University, Wuxi 214126, China; 4 APC Microbiome Ireland, University College Cork, T12 K8AF Cork, Ireland; 5 Teagasc Food Research Centre, Moorepark, Fermoy, P61 C996 Cork, Ireland; 6 National Engineering Research Center for Functional Food, Jiangnan University, Wuxi 214126, China

**Keywords:** *Bifidobacterium breve*, psoriasis, gut microbiota, dose–response efficacy, bile acids, FXR/NF-κB pathway

## Abstract

This study aimed to investigate the dose–response effect of *Bifidobacterium breve* CCFM683 on relieving psoriasis and its underlying patterns. Specifically, the expression of keratin 16, keratin 17, and involucrin were substantially decreased by administration of 10^9^ CFU and 10^10^ CFU per day. Moreover, interleukin (IL)-17 and TNF-α levels were substantially decreased by 10^9^ and 10^10^ CFU/day. Furthermore, the gut microbiota in mice treated with 10^9^ or 10^10^ CFU/day was rebalanced by improving the diversity, regulating microbe interactions, increasing *Lachnoclostridium*, and decreasing *Oscillibacter*. Moreover, the concentrations of colonic bile acids were positively correlated with the effectiveness of the strain in relieving psoriasis. The gavage dose should be more than 10^8.42^ CFU/day to improve psoriasis according to the dose–effect curve. In conclusion, CCFM683 supplementation alleviated psoriasis in a dose-dependent manner by recovering microbiota, promoting bile acid production, regulating the FXR/NF-κB pathway, diminishing proinflammatory cytokines, regulating keratinocytes, and maintaining the epidermal barrier function. These results may help guide probiotic product development and clinical trials in psoriasis.

## 1. Introduction

Psoriasis is an immune-mediated systemic disease characterized by keratinocyte hyperproliferation and differentiation dysfunction in the epidermis [1]. Erythema and scaling of the skin are regarded as the typical symptoms of psoriasis. Allopathic remedies including corticosteroids, vitamin D analogs, methotrexate, and UV phototherapy may have side effects such as cutaneous atrophy, dyspigmentation, or bone marrow toxicity, thus limiting their application [2]. New therapies such as probiotics and prebiotics are used to treat psoriasis via modulating gut microbiota and the host immune response, which is a substitute for allopathic remedies [3,4].

*Lactiplantibacillus plantarum* GMNL-77 (2 × 10^9^ CFU/day) has been reported to decrease the proportion of IL-17A+CD4+T cells and reduce IL-23 and IL-17, thus relieving psoriasis [5]. *B. adolescentis* CCFM667, *Lacticaseibacillus paracasei* CCFM1074, and *Limosilactobacillus reuteri* CCFM1132 (5 × 10^9^ CFU/day) were found to decrease the relative abundance of *Rikenellaceae*, thus recovering the unbalanced gut microbiota in psoriasis mice [6]. *Leuconostoc mesenteroides* NTM048 alleviated psoriasis (1 × 10^10^ CFU/day) via diminishing IL-17 and its receptor [7]. Other probiotic preparations, including *Staphylococcus epidermidis* ATCC12228 (extracellular vesicles), *Lactilactobacillus sakei* proBio-65 (ethanol extract), and Se-rich brewer’s yeast (peptide fraction) ameliorate psoriasis by inhibiting the NF-κB pathway or reducing proinflammatory cytokines [8,9,10]. Moreover, 3 × 10^6^ CFU/day *Lactobacillus rhamnosus* ATCC7469, 1 × 10^8^ CFU/day *Streptococcus salivarius* K12, 1 × 10^10^ CFU/day *B. infantis* 35624, and the probiotic mixture containing nine bacterial strains of *Lactobacillus* and *Bifidobacterium* with at least 7.5 × 10^8^ CFU/portion decreased PASI in psoriasis patients [11,12,13,14]. Thus, the effects of probiotics on psoriasis were confirmed but species- even strain-specific. Additionally, the effectiveness of live bacteria in psoriasis alleviation requires an adequate dosage, which lies between 10^8^ and 10^10^ CFU/day.

An “adequate amount” is necessary for probiotics to beneficially act on the host. However, the specific dose of this “adequate amount” is not commonly specified [15]. The efficacious dose depends on various factors including the specific strain, formulation, and its probiotic functions. In our preliminary study, *B. breve* CCFM683 showed psoriasis-relieving effects in mice. However, the effective dose for CCFM683 to relieve psoriasis remains unclear.

In the current study, we explored the dose–response effect of psoriasis alleviation by *B. breve* CCFM683 and obtained its optimal gavage dose by curve fitting. Moreover, the impact of gavage doses on the crucial links for CCFM683 to ameliorate psoriasis was investigated. These results will reveal the relationships between gavage dosage of CCFM683, colonic bile acid concentrations, FXR/NF-κB expression, and psoriasis remission. It may help to guide probiotic product development and clinical trials in psoriasis.

## 2. Materials and Methods

### 2.1. Strain Culture Conditions

*B. breve* CCFM683 was obtained from the Culture Collection of Food Microorganisms (CCFM) in Jiangnan University (Wuxi, China). The strain was sub-cultured as previously described before gavage [16]. CCFM683 cell pellets were obtained by centrifuging at 6000× *g* and then they were diluted with sterile saline to the concentration of 5 × 10^6^, 5 × 10^7^, 5 × 10^8^, 5 × 10^9^, and 5 × 10^10^ CFU/mL before administration.

### 2.2. Animal Experiment Design

Female Balb/c mice (6-week-old, 16–18 g) were purchased from Vital River Laboratory Animal Technology Co., Ltd. (Beijing, China) and adapted for 7 days before experiments. The animals were maintained in the barrier facility of Jiangnan University at a constant temperature of 20 ± 2 °C, a humidity of 50 ± 5%, and a photological cycle of 12/12 h darkness, and were fed sterile water and commercial chow.

The psoriasis in mice was induced following a previous description [6]. The experimental protocols are shown in Table 1. Specifically, mice were randomly divided into 8 groups (*n* = 8): control, imiquimod (IMQ), methotrexate (MTX), 10^6^ CFU/day CCFM683, 10^7^ CFU/day CCFM683, 10^8^ CFU/day CCFM683, 10^9^ CFU/day CCFM683, and 10^10^ CFU/day CCFM683. An amount of 62.5 mg imiquimod (3M Pharmaceuticals, St. Paul, MN, USA) was applied to the dorsal skin after shaving and 20 mg was applied to the right ear on the 15th–20th days to induce psoriasis in mice. Mice in the MTX group were gavaged with 1 mg/mL methotrexate (SPH Sine Pharmaceutical Laboratories, Shanghai, China) dissolved in 0.9% saline; 0.85% saline was administered to mice in the control and IMQ groups; and 0.2 mL 5 × 10^6^, 5 × 10^7^, 5 × 10^8^, 5 × 10^9^, or 5 × 10^10^ CFU/mL of *B. breve* CCFM683 were orally administered to the mice in each group correspondingly. The animal experiment was under the supervision of the Experimental Animal Ethics Committee of Jiangnan University (qualified number: JN.No20220615b0880807[189]).

### 2.3. Assessment of Psoriasis

The ear thickness and body weight were measured daily during the IMQ application, and the weight percentage relative to the initial weight was calculated. The clinical psoriasis area and severity index (PASI) was assessed daily to evaluate the degree of thickening, scaling, and erythema by the following system [17]: 0 (none), 1 (mild), 2 (moderate), 3 (marked), and 4 (very marked). The three components added up to the total PASI score.

Mice in all groups were sacrificed after fasting for 12 h on the 20th day, and the serum was obtained by centrifuging the blood samples at 3000× *g* for 20 min. The spleen weight was measured, and 4% paraformaldehyde was used to fix the dorsal skin tissue. The fixed skin tissue was stained by hematoxylin and eosin (H&E) according to a previous description [16]. Hyperkeratosis, parakeratosis, necrosis, and thickening in the epidermis along with hypertrophy and inflammatory infiltration in the dermis were evaluated according to the following system with proper modification [17]: 0 (none), 1 (mild), 2 (moderate), and 3 (marked). The components above added up to the total histopathological score.

### 2.4. Biochemical Assays

The skin tissue supernatant was collected as previously described [18]. Briefly, skin tissue ground with RIPA buffer (Beyotime Biotechnology, Shanghai, China) was centrifuged at 12,000× *g* for 10 min to obtain the supernatant. ELISA kits (R&D Systems, Minneapolis, MN, USA) were used to determine the cutaneous IL-17, TNF-α, IL-1β, and IL-6, and a BCA Protein ELISA kit (Beyotime Biotechnology, Shanghai, China) was used to quantify the concentration of total cutaneous proteins.

### 2.5. Quantitative RT-PCR

Cutaneous total RNA extracted with TRIzol reagent (Vazyme, Nanjing, China) was used to generate complementary DNA obtained by reverse transcription with commercial kits (Takara, Tokyo, Japan). Quantitative PCR was carried out using an RT-PCR System (Bio-Rad, Hercules, CA, USA). The gene expression was quantified with the method of 2^−ΔΔCt^. The primers of the determined genes are shown in Table 2.

### 2.6. Protein Expression Determination

The protein level of proliferating cell nuclear antigen (PCNA), farnesoid X receptor (FXR), p65, phosphor-p65 (*p*-p65), IκB, and phosphor-IκB (*p*-IκB) in the skin were measured by Western blot assays which were carried out following a previous description [19]. In brief, skin tissue lysed with RIPA buffer was centrifuged at 12,000× *g* to obtain the supernatant and the proteins in the supernatant were separated using electrophoresis with 8–12% SDS–polyacrylamide gel and transferred onto the PVDF membranes. Primary antibodies (Abcam, Cambridge, UK) were incubated with the membranes which had been blocked with 2% BSA for 2 h. Then, a secondary anti-rabbit or anti-mouse IgG antibody (Abcam, Cambridge, UK) was incubated with the membranes. The protein content represented by the band density was determined with Image J2 (Bethesda, MD, USA).

### 2.7. Bile Acid Analysis

Colonic bile acid extraction was performed as previously described with proper modification [20]. Briefly, colonic content (20 mg) was homogenized in 400 μL methanol and centrifuged at 12,000× *g* and 4 °C for 15 min. The supernatant was collected and evaporated in a centrifuge (Eppendorf, Hamburg, Germany) to remove the solvent. The obtained solute was resolved in 400 μL methanol and centrifuged under the same conditions before use. Bile acid standards (Sigma-Aldrich, St. Louis, MO, USA), including deoxycholic acid (DCA), ursodeoxycholic acid (UDCA), tauroursodeoxycholic acid (TUDCA), hyodeoxycholic (HDCA), lithocholic acid (LCA), β-muricholic acid (β-MCA), taurocholic acid (TCA), and cholic acid (CA), were used as internal standards. A UPLC-MS system (Thermo Fisher Scientific, Waltham, MA, USA) was used to analyze the prepared samples. The instrumental settings were set and data processing was performed as previously described [21].

### 2.8. Fecal DNA Sequencing and Bioinformatics Analysis

Fecal samples of each mouse were collected with sterile tweezers in individual Eppendorf tubes on the 20th day before fasting and sacrificing. The FastDNA Spin Kit (MP Biomedicals, Irvine, CA, USA) was used to extract fecal DNA, the V3–V4 region of which was amplified using Premix TaqTM (CoWin Biosciences, Yancheng, China) with the primers 341F and 806R [18]. A commercial gel extraction kit (Biomiga, San Diego, CA, USA) and a Qubit™ 4 Fluorometer (Life Technologies, South San Francisco, CA, USA) were used to purify and quantified the obtained PCR products. Library construction, sequencing, and QIIME pipeline analysis were performed according to an earlier description [18].

The analyses of α- and β-diversity were conducted online (https://www.microbiomeanalyst.ca/, accessed on 10 January 2023) [22]. The proportions of specific genera and the significant differences among them were revealed with STAMP (v 2.1.3) [23]. An RMT-based network analysis was used to evaluate each OTUs topological role. A correlation analysis of the psoriatic symptoms and the proportions of specific genera was conducted with Pearson analysis.

### 2.9. Statistical Analysis

The data were analyzed with SPSS 22.0 and GraphPad Prism 8.0. A one-way ANOVA according to Tukey’s tests was used to analyze the significant differences which were represented by the *p*-value.

## 3. Results

### 3.1. The Effect of B. breve CCFM683 on Psoriasis Symptoms

**Figure 1:**
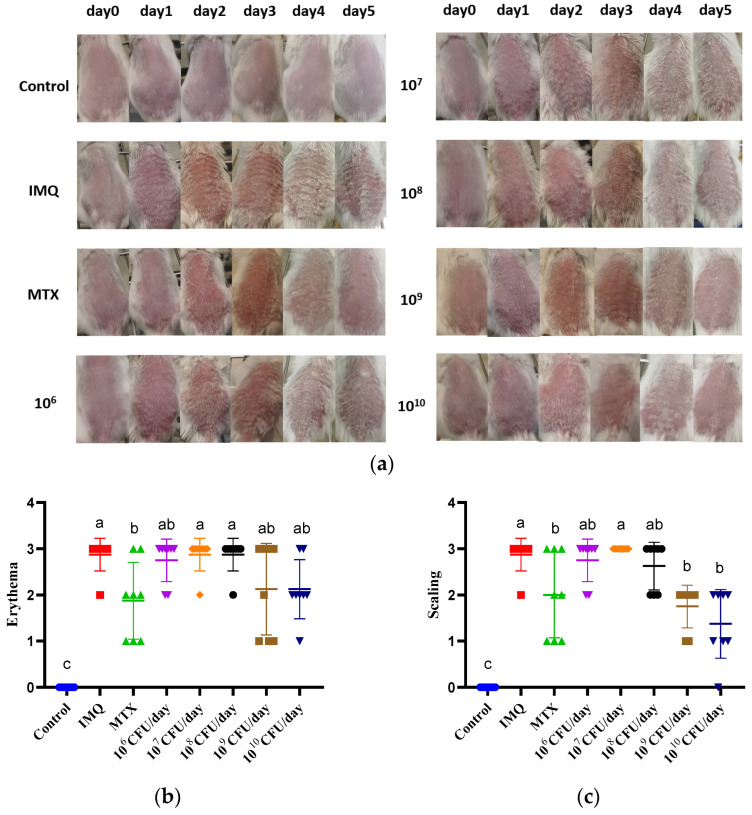

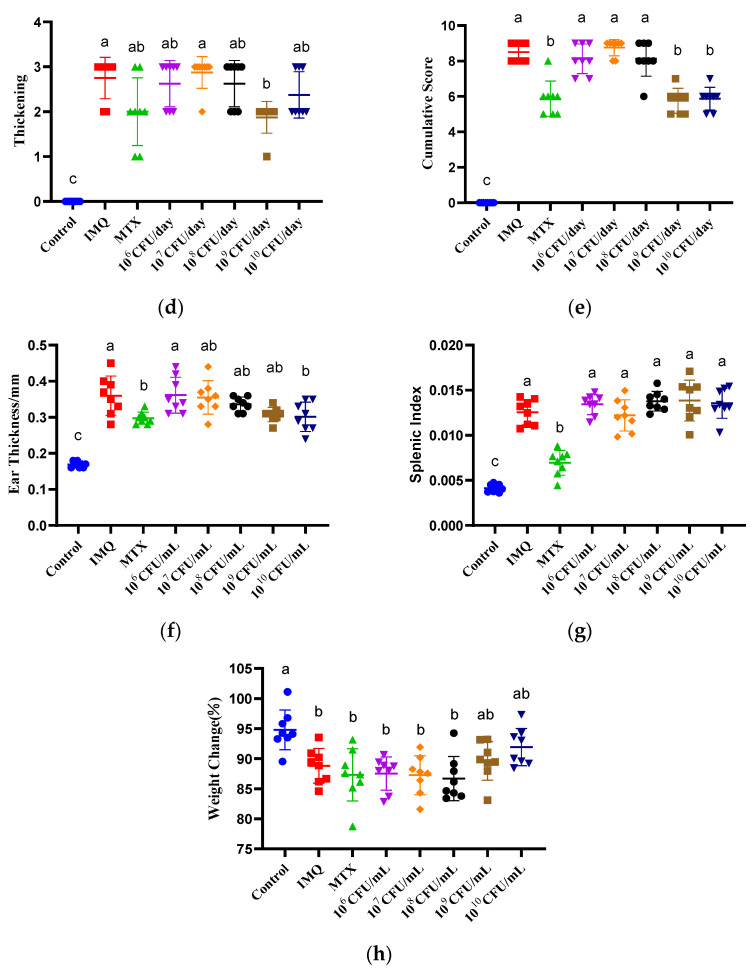
Effect of CCFM683 treatment on IMQ-induced psoriasis. (**a**) Macroscopic pictures of skin lesions. (**b**) Erythema, (**c**) scaling, (**d**) thickening, (**e**) ear thickness, (**f**) cumulative score, (**g**) splenic index, and (**h**) change in body weight (%). *n* = 8 mice per group. Groups with different letters are significantly (*p* < 0.05) different from each other.

The protective effect of *B. breve* CCFM683 at different doses (10^6^ to 10^10^ CFU/day) on psoriasis in mice was evaluated to explore its dose–response efficacy. Treatment with 10^9^ and 10^10^ CFU/day CCFM683 and MTX showed protective effects to different extents (Figure 1a). Specifically, 10^9^ and 10^10^ CFU/day CCFM683 prevented ear thickening by 14.4% and 16.4%, respectively, whereas MTX reduced ear thickness by 17.2% compared with the IMQ-treated mice (*p* < 0.05) (Figure 1b). The cumulative score of 10^9^ CFU/day CCFM683-, 10^10^ CFU/day CCFM683-, and MTX-treated mice were, respectively, 67.6%, 69.1%, and 69.1% of that of the IMQ-treated mice (*p* < 0.05) (Figure 1c). MTX treatment led to a reduction in splenic weight (*p* < 0.05), while 10^9^ and 10^10^ CFU/day CCFM683 had no difference compared with the IMQ group (Figure 1d). Additionally, IMQ treatment caused weight loss in mice, which could not be ameliorated by either MTX or 10^9^ CFU/day CCFM683 (Figure 1e). Treatment with 10^9^ and 10^10^ CFU/day CCFM683, although without significance, reduced the weight loss caused by IMQ exposure. Treatment with 10^6^, 10^7^, or 10^8^ CFU/day CCFM683 showed no obvious protective effects on the above symptoms.

### 3.2. The Effect of B. breve CCFM683 on Histological Characteristics in Psoriasis Mice

**Figure 2:**
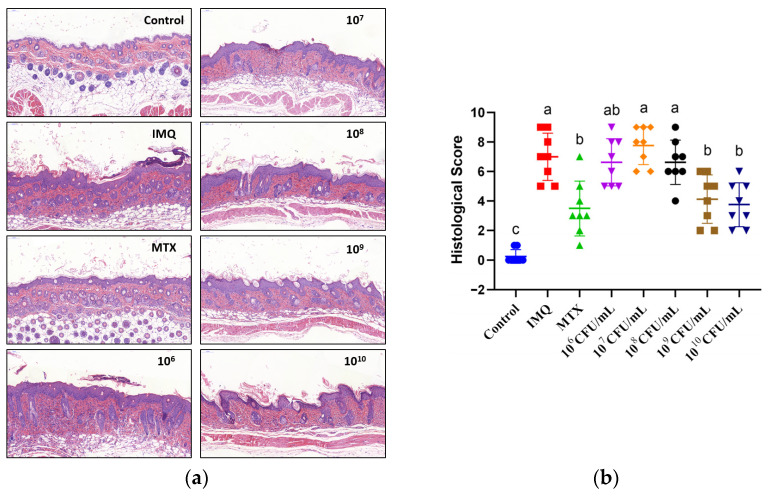
Effect of CCFM683 treatment on histological characteristics in psoriasis mice. (**a**) Cutaneous histology and (**b**) histological score of skin. *n* = 8 mice per group. Groups with different letters are significantly (*p* < 0.05) different from each other.

H&E staining was performed to assess the histological characteristics of dorsal skin. Mice in the control group had an intact epidermis and a thin corneum, and no hypertrophy or inflammatory infiltration was found, whereas the mice of the IMQ group showed a necrotic epidermis, an exfoliated corneum, and a massive amount of inflammatory cells (Figure 2a). In the histopathological assessment, the control mice scored only 3.5% of the value of the IMQ group (Figure 2b). The histological scores in 10^9^ CFU/day CCFM683-, 10^10^ CFU/day CCFM683-, and MTX-treated mice decreased by 41.1%, 46.4, and 50.0%, respectively, in comparison with that of the IMQ group (*p* < 0.05). No substantial decline was found in the histological score of the 10^6^, 10^7^, or 10^8^ CFU/day CCFM683-treated mice. Therefore, an adequate dose between 10^9^ and 10^10^ CFU/day might be necessary for *B. breve* CCFM683 to ameliorate psoriasis in mice.

### 3.3. Dose–Effect Curve between Gavage Doses and Psoriasis Remission

**Figure 3:**
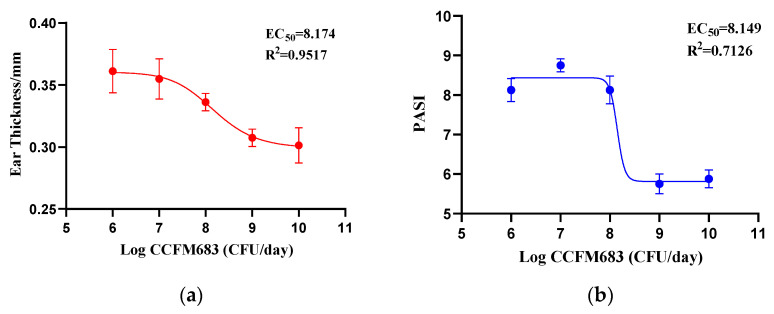

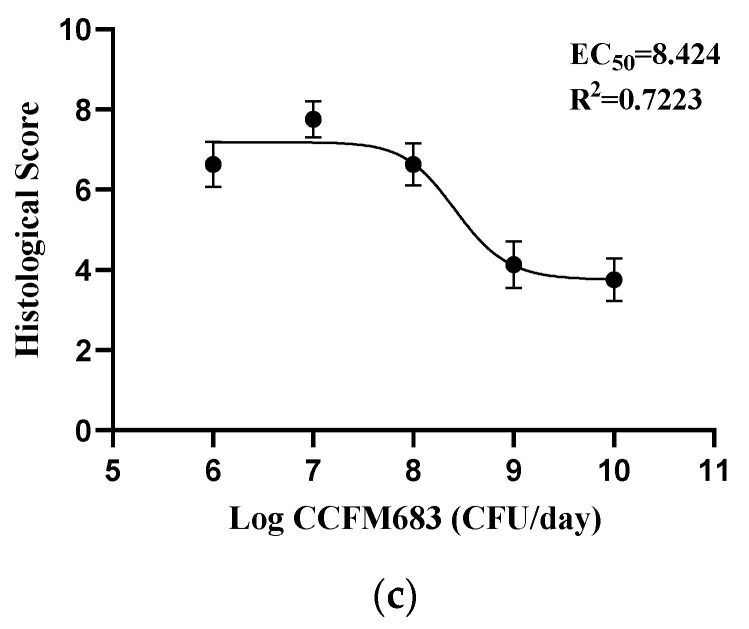
The dose–effect curve between CCFM683 dose and psoriasis indicators. (**a**) Ear thickness, (**b**) cumulative score, and (**c**) histological score of the skin. *n* = 8 mice per group.

Dose–response curves between gavage dose and ear thickness, cumulative score, and histological score were generated to explore the relationship between the administration amount of CCFM683 and psoriasis amelioration. The S-shaped curves of the gavage dose and psoriasis indexes indicated the dose–response efficacy of CCFM683 in relieving psoriasis (Figure 3a–c). The EC50 between the gavage dose and histological score was 10^8.42^ CFU/day. Moreover, the EC50 between the gavage dose and ear thickness and PASI were 10^8.17^ CFU/day and 10^8.15^ CFU/day, respectively. As the cutaneous histological score is the key indicator of psoriasis development, it could be inferred that a gavage dose of more than 10^8.42^ CFU/day was necessary for CCFM683 to ameliorate psoriasis in mice.

### 3.4. The Effect of B. breve CCFM683 on Keratinocytes and Epidermal Barrier in Psoriasis Mice

**Figure 4:**
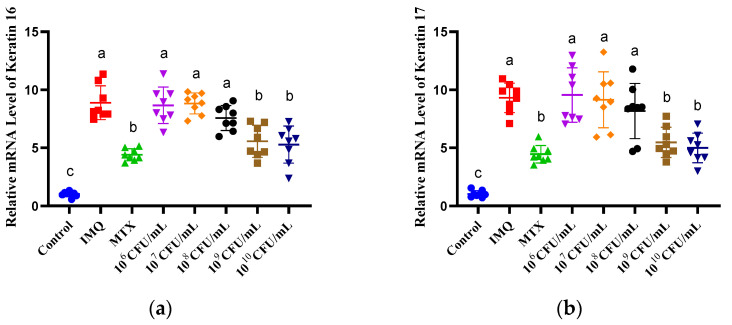

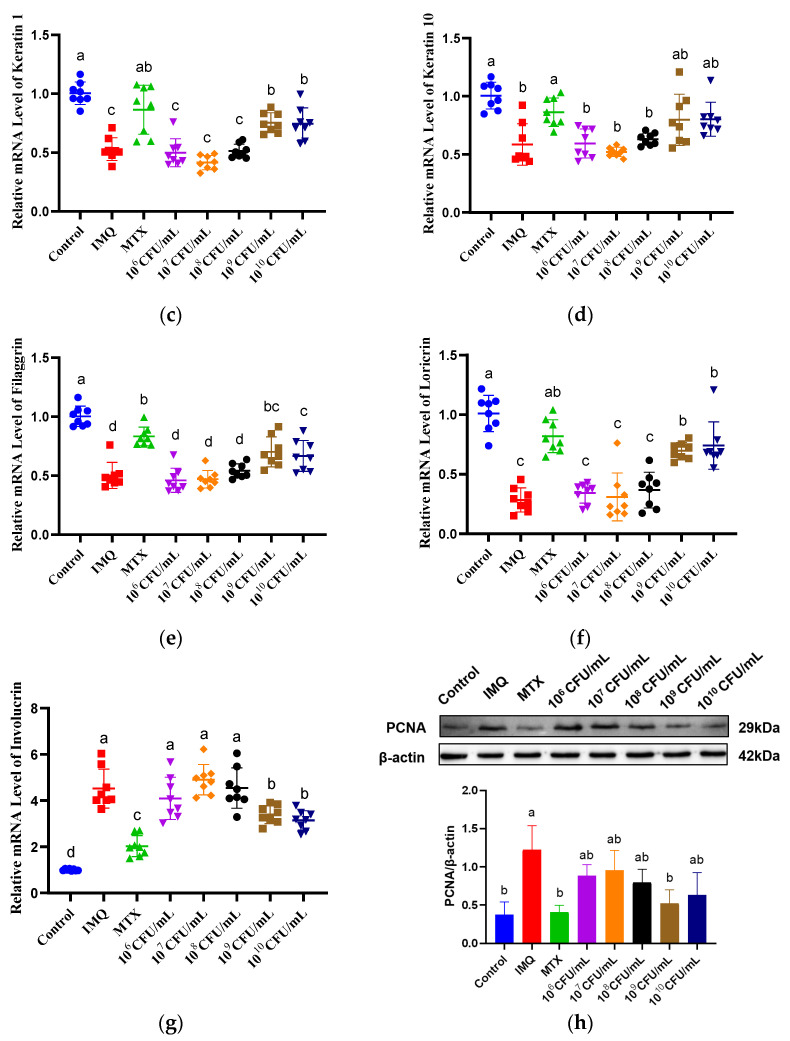
Effect of CCFM683 treatment on keratinocytes and epidermal barrier in psoriasis mice. Relative mRNA levels of (**a**) keratin 16, (**b**) keratin 17, (**c**) keratin 1, (**d**) keratin 10, (**e**) filaggrin, (**f**) loricrin, and (**g**) involucrin, and (**h**) protein levels of PCNA. *n* = 8 mice per group in mRNA determination and *n* = 3 mice per group for protein measurement. Groups with different letters are significantly (*p* < 0.05) different from each other.

The mRNA level of the keratins (keratin 1, keratin 10, keratin 16, and keratin 17) and proliferating cell nuclear antigen (PCNA) were determined to assess the effects of *B. breve* CCFM683 on keratinocytes. IMQ exposure increased keratin 16 and 17 (Figure 4a,b) and decreased keratin 1 and 10 (Figure 4c,d) compared with those of the control. Treatment with 10^9^ CFU/day CCFM683, 10^10^ CFU/day CCFM683, and MTX increased keratin 1 and keratin 10 and reduced keratin 16 and keratin 17 (*p* < 0.05). PCNA was rarely expressed in the control group and increased with IMQ exposure (Figure 4h). After the MTX or 10^9^ CFU/day CCFM683 treatment, PCNA was reduced to a similar level to that of the control mice (*p* < 0.05). Treatment with 10^10^ CFU/day CCFM683 induced a reduction in PCNA, but without significance (*p* = 0.06).

To evaluate the effect of CCFM683 on the epidermal barrier, the mRNA levels of involucrin, loricrin, and filaggrin were determined. The expression of loricrin and filaggrin was inhibited by IMQ application and elevated after 10^9^ CFU/day CCFM683, 10^10^ CFU/day CCFM683, or MTX treatment (*p* < 0.05) (Figure 4e,f). Involucrin was rarely expressed in the dorsal skin of the control mice, which was increased by IMQ exposure (Figure 4g). After the treatment with 10^9^ CFU/day CCFM683, 10^10^ CFU/day CCFM683, or MTX, involucrin was substantially down-regulated in comparison with that of the IMQ group (*p* < 0.05). On the contrary, 10^6^, 10^7^, and 10^8^ CFU/day CCFM683 treatment had no influence on either keratinocytes or the epidermal barrier. This was consistent with the curve fitting result that more than 10^8.42^ CFU/day was required for CCFM683 to ameliorate psoriasis.

### 3.5. The Effect of B. breve CCFM683 on Bile Acid Metabolism in Psoriasis Mice

**Figure 5:**
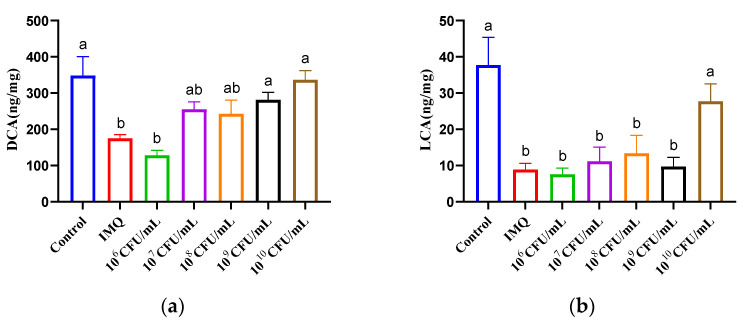
Effect of CCFM683 treatment on colonic bile acids in psoriasis mice. (**a**) DCA and (**b**) LCA in the colon. *n* = 8 mice per group. Groups with different letters are significantly (*p* < 0.05) different from each other.

The bile acid metabolism influences psoriasis development, and certain secondary bile acids has been confirmed to alleviate psoriasis. Here, we determined the concentration of the bile acids in the colon to evaluate the effect of CCFM683 doses on bile acid metabolism. DCA and LCA were both reduced in psoriasis mice compared with control mice (Figure 5a,b). Treatment with 10^10^ CFU/day CCFM683 significantly increased colonic DCA and LCA compared with the IMQ group (*p* < 0.05), whereas 10^9^ CFU/day CCFM683 substantially elevated DCA. However, the other three doses of CCFM683 treatments induced no significant difference in either bile acid. In fact, after being treated with CCFM683 at the dose of 10^9^ or 10^10^ CFU/day, UDCA, TUDCA, HDCA, β-MCA, TCA, and CA in mice were higher than those of the IMQ group, but without significance (Figure S1a–f).

**Figure 6:**
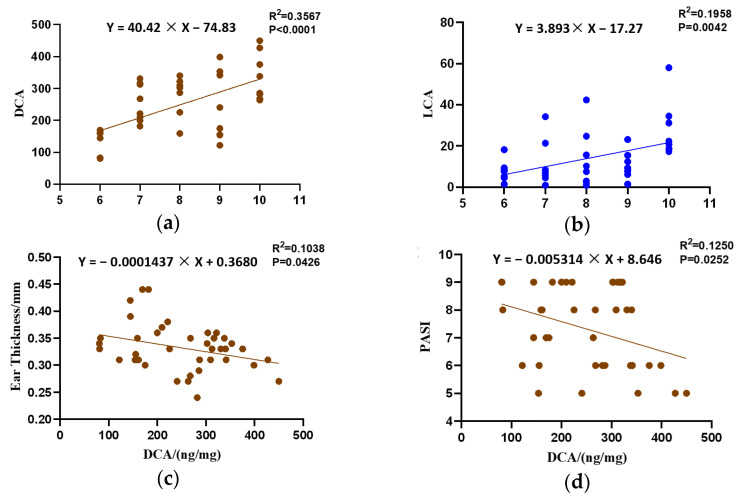
Quantitative relationships between the colonic bile acid concentrations and psoriasis indicators. The interdependent quantitative relationships between the CCFM683 doses and colonic (**a**) DCA and (**b**) LCA concentrations. Quantitative relationships between the DCA concentration and (**c**) ear thickness and (**d**) cumulative score.

A correlation analysis illustrated that the administration doses of CCFM683 were positively correlated with the colonic DCA and LCA (*p* < 0.05) (Figure 6a,b). Moreover, the concentration of colonic DCA was negatively correlated with ear thickness and cumulative score (*p* < 0.05) (Figure 6c,d). In fact, the colonic LCA concentration was also negatively correlated with psoriasis indicators, but without significance (Figure S2a–c). This implies that DCA and LCA resulted from the CCFM683 gavage and might play an important role in psoriasis alleviation.

### 3.6. The Effect of B. breve CCFM683 on FXR/NF-κB Pathway and Immune Responses in Psoriasis

**Figure 7:**
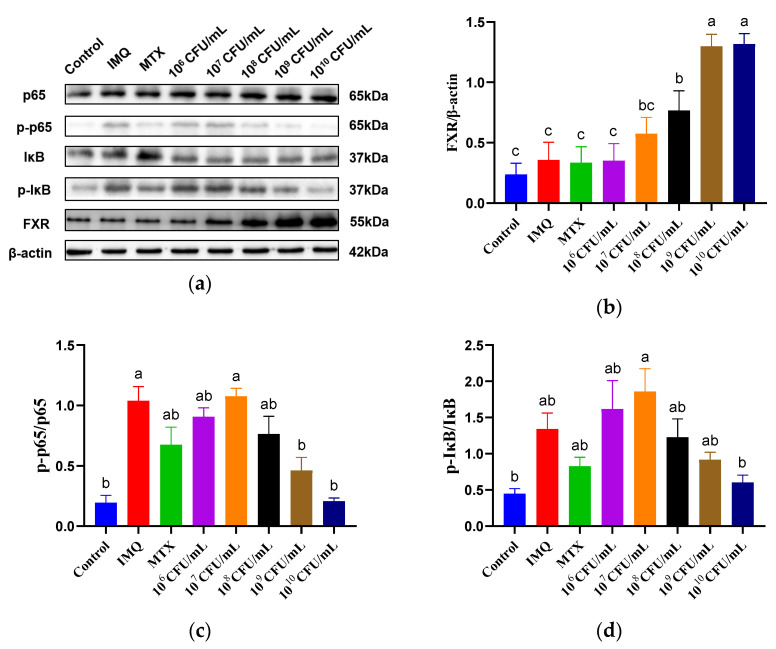
Effect of CCFM683 treatment on the FXR /NF-κB pathway in psoriasis mice. (**a**–**d**) Quantitative analysis of the key proteins in FXR/NF-κB pathway in IMQ-treated mice by Western blotting. *n* = 3 mice per group. Groups with different letters are significantly (*p* < 0.05) different from each other.

As shown above, CCFM683 had an obvious dose-dependent effect on bile acid production and psoriasis alleviation. To explore whether the bile-acid-related signaling pathway depends on the gavage dose, the bile acid receptor FXR and the principal proteins in the NF-κB pathway were determined by Western blot assays (Figure 7a). MTX had no influence on the protein level of FXR, whereas 10^9^ and 10^10^ CFU/day CCFM683 showed predominant activation (*p* < 0.05) (Figure 7b). This was consistent with the remission effect and bile acid production of CCFM683 at various doses in psoriasis. Interestingly, 10^8^ CFU/day CCFM683, although not as much as 10^9^ or 10^10^ CFU/day CCFM683, also elevated the expression of FXR (*p* < 0.05). This implies that the activating effect of 10^8^ CFU/day CCFM683 on FXR was significant but unable to improve psoriasis. It has been reported that FXR activation suppresses the expression of the NF-κB in various diseases, and NF-κB was strictly required for psoriasis progression. Correspondingly, the phosphorylation of p65 and IκB in IMQ-treated mice was 5.33 and 2.98 times higher than those in control mice and was recovered by 10^10^ CFU/day CCFM683 treatment (*p* < 0.05) (Figure 7c,d). Treatment with 10^9^ CFU/day CCFM683 also reduced, although not significantly, *p*-p65 and *p*-IκB (*p* = 0.018 and *p* = 0.882, respectively). Doses of 10^6^, 10^7^, and 10^8^ CFU/day had no influence on the expression of the FXR/NF-κB pathway, probably because they failed to increase the bile acids to adequate concentrations in the colon.

**Figure 8:**
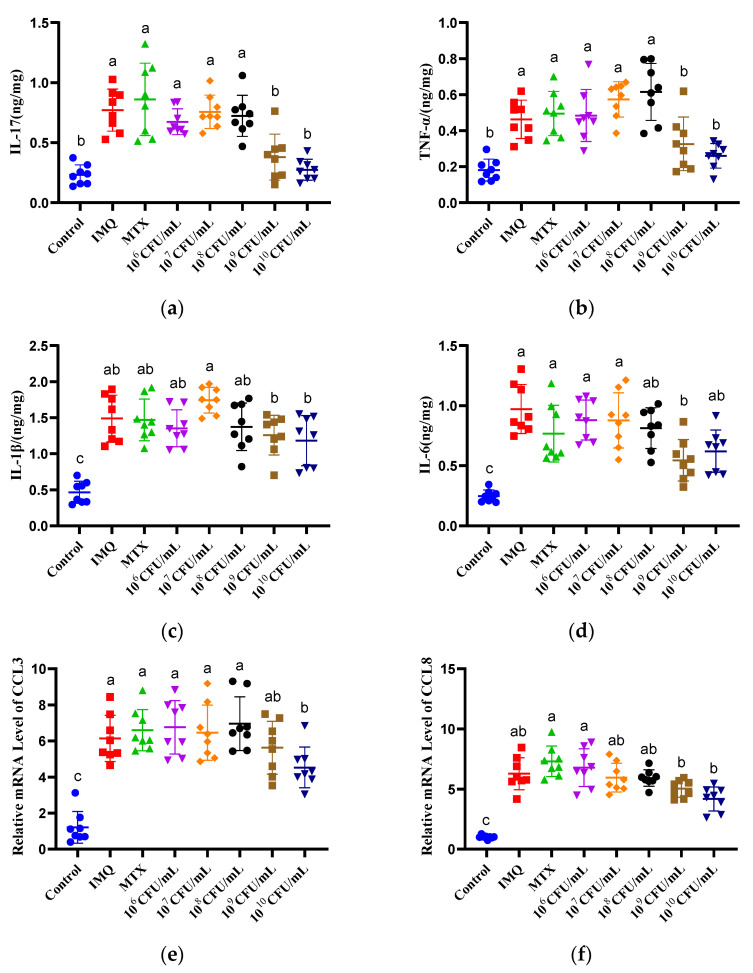

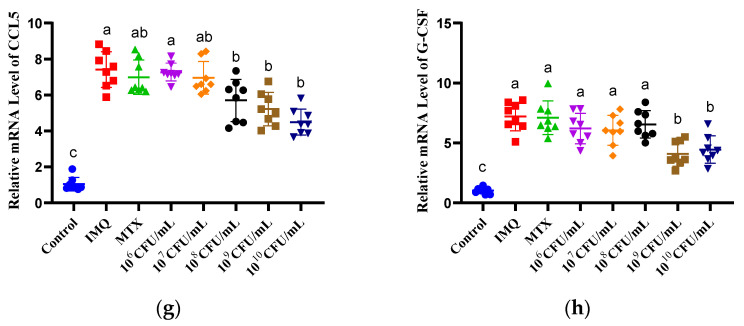
Effect of CCFM683 treatment on inflammatory responses in psoriasis mice. The concentration of (**a**) IL-17, (**b**) TNF-α, (**c**) IL-1β, and (**d**) IL-6 and the mRNA levels of (**e**) CCL3, (**f**) CCL8, (**g**) CCL5, and (**h**) G-CSF. *n* = 8 mice per group. Groups with different letters are significantly (*p* < 0.05) different from each other.

The cytokines driven by NF-κB activation in psoriasis, including IL-17, TNF-α, IL-1β, and IL-6, were increased by IMQ exposure (*p* < 0.05) (Figure 8a–d). Treatment with 10^9^ and 10^10^ CFU/day CCFM683 significantly diminished these four cytokines (*p* < 0.05). Moreover, the mRNA levels of CCL3, CCL5, CCL8, and G-CSF in mice treated with 10^10^ CFU/day CCFM683 were 73.72%, 60.64%, 66.50%, and 61.85% of those in the IMQ group, respectively (Figure 8e–h). Treatment with 10^9^ CFU/day CCFM683 substantially inhibited the expression of CCL5 and G-CSF (*p* < 0.05). No inhibiting effect on any cytokine was found in the 10^6^, 10^7^, or 10^8^ CFU/day CCFM683 groups. Notably, no cytokine was decreased by MTX either, which indicated that MTX helped inhibit hyperproliferation but rarely relieved immune disorders in psoriasis mice.

### 3.7. The Effect of B. breve CCFM683 on Gut Microbiota

**Figure 9:**
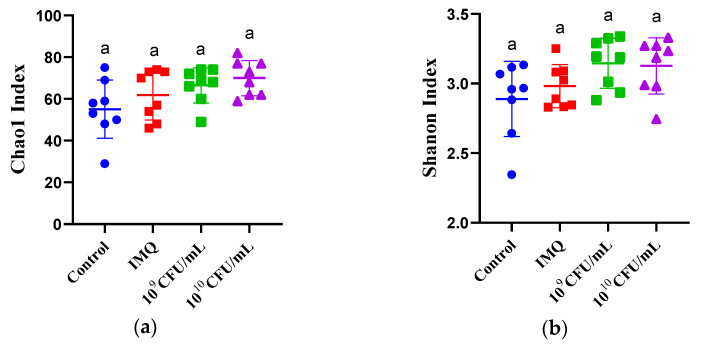

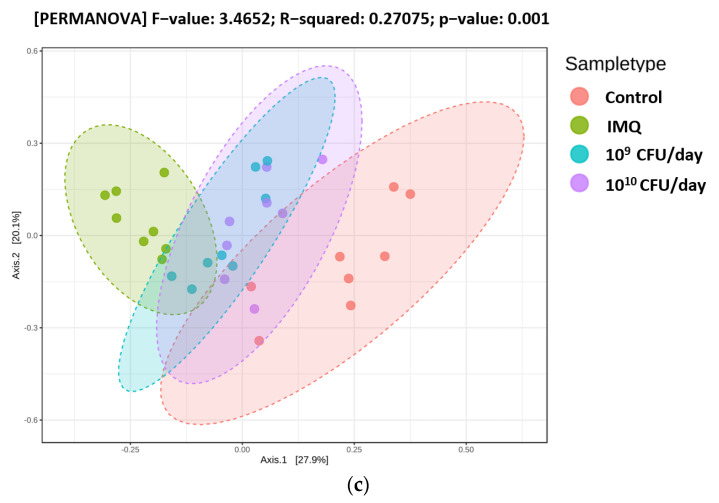
Effect of CCFM683 treatment on the gut microbial diversity in psoriasis mice. The α-diversity includes (**a**) Chao1 and (**b**) Shannon indices. (**c**) PCoA comparing the microbiota structure of CCFM683-treated mice. Groups with different letters are significantly (*p* < 0.05) different from each other.

The α-diversity of gut microbiota was represented by Chao1 and Shannon indexes, which were both elevated in 10^9^ and 10^10^ CFU/day CCFM683-treated mice compared with IMQ-treated mice, but not significantly (*p* = 0.47, *p* = 0.91, *p* = 0.61, and *p* = 0.49, respectively) (Figure 9a,b). PCoA based on the Bray–Curtis distance matrices calculated by PERMANOVA was used to represent the β-diversity. The results showed that the gut microbiota of the control and IMQ-treated mice were significantly different and divided from each other (Figure 9c). Treatment with 10^9^ or 10^10^ CFU/day CCFM683 partly reversed the shift caused by IMQ exposure.

**Figure 10:**
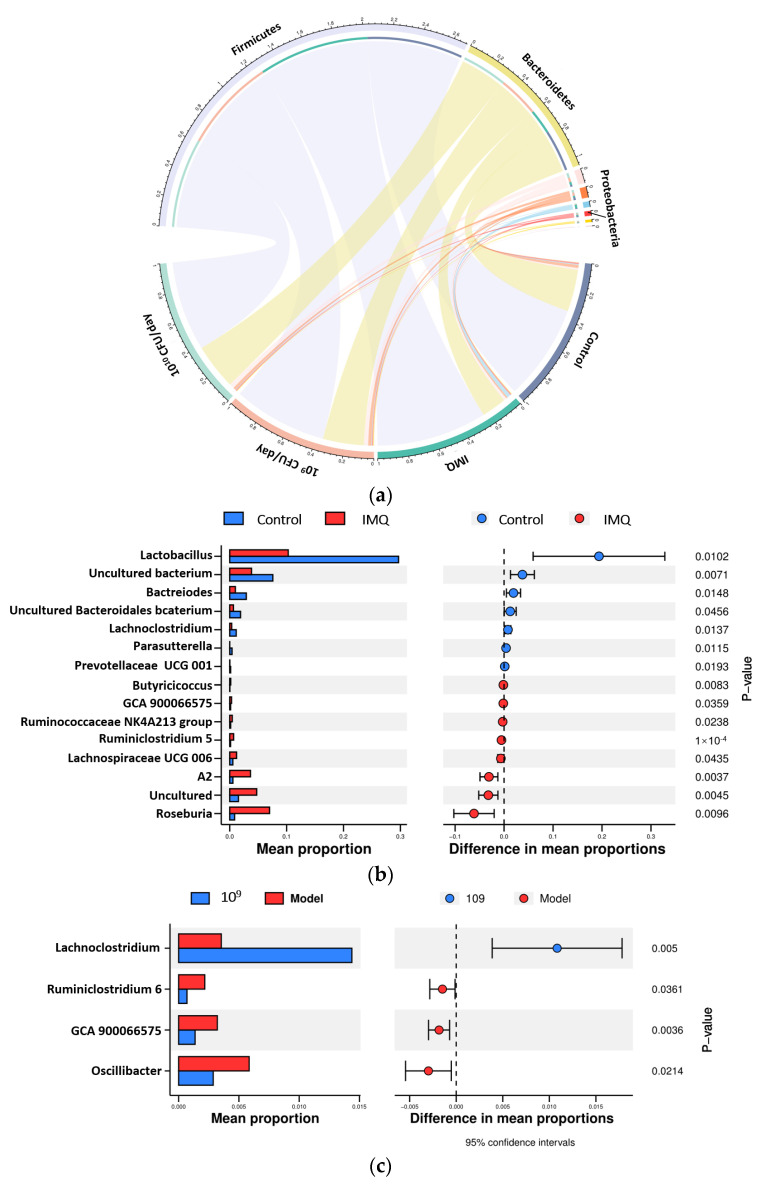

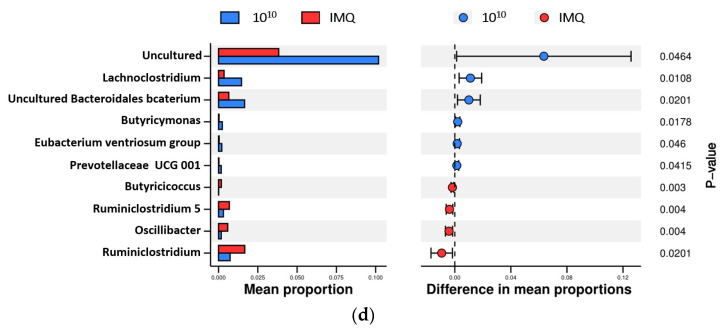
Effect of CCFM683 treatment on the gut microbial composition in IMQ-treated mice. (**a**) Phylum level of gut microbiota. Genus level of microbiota in control vs. IMQ (**b**), 10^9^ CFU/day CCFM683 vs. IMQ (**c**), and 10^10^ CFU/day CCFM683 vs. IMQ groups (**d**) were compared.

The microbial composition was analyzed to further evaluate the effect of CCFM683 on the gut microbiota. In control mice, Firmicutes (66.06%), Bacteroidetes (29.05%), Patescibacteria (1.28%), and Proteobacteria (0.72%) were predominant (Figure 10a). Nevertheless, in the IMQ-treated mice, the relative abundances of Firmicutes and Proteobacteria rose to 76.07% and 0.83%, respectively, whereas Bacteroidetes decreased to 17.14%. Treatment with 10^9^ and 10^10^ CFU/day CCFM683 induced a decrease in the proportion of Firmicutes and Proteobacteria as well as an increase in the proportion of Bacteroidetes. Additionally, the relative abundance of Deferribacteres was increased after treatment with 10^9^ or 10^10^ CFU/day CCFM683.

To further evaluate the influence of IMQ and CCFM683 treatment on the structure of the gut microbiota in mice, a Welch’s *t*-test was employed to identify the substantially altered genera. IMQ application remarkably diminished the relative abundance of the *Lactobacillus*, *Bacteroides*, *Lachnoclostridium*, *Parasutterella*, and *Prevotellaceae UCG 001*, whereas the relative abundances of *Butyricicoccus*, *GCA 900066575*, the *Ruminococcaceae NK4A214 group*, *Ruminiclostridium 5*, *Lachnospiraceae UCG 006*, *A2*, and *Roseburia* were significantly elevated (*p* < 0.05) (Figure 10b). Both 10^9^ and 10^10^ CFU/day CCFM683 treatments drastically elevated *Lachnoclostridium* and diminished *Oscillibacter* compared with the IMQ-treated mice (Figure 10c,d).

**Figure 11:**
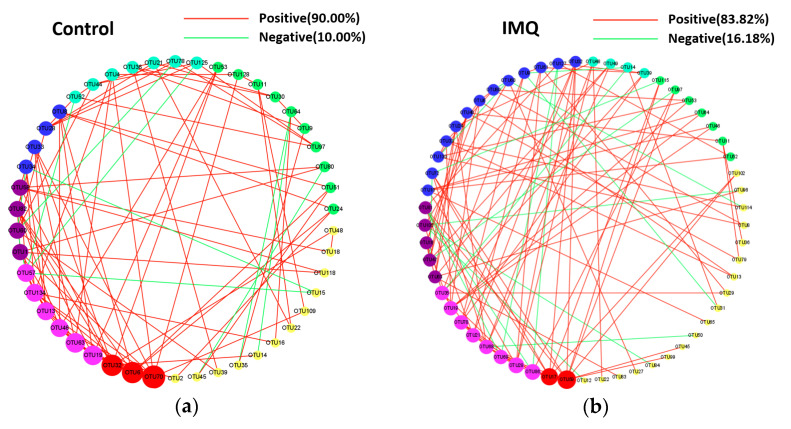

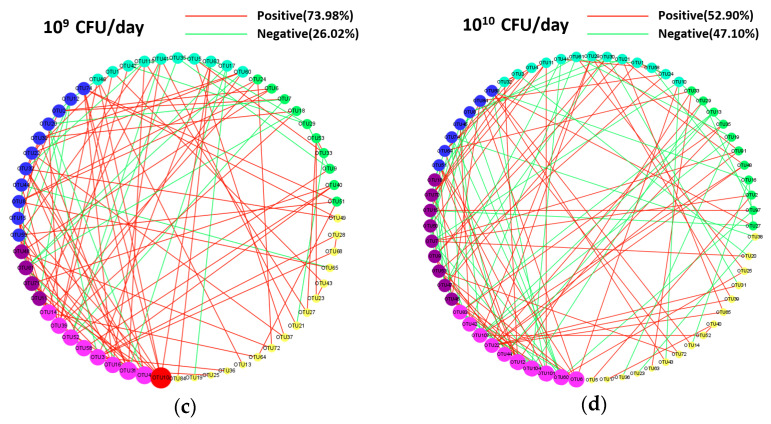
Effects of CCFM683 treatment on dominant microorganisms. The co-occurrence network structure of gut bacteria in (**a**) control, (**b**) IMQ, (**c**) 10^9^ CFU/day CCFM683, and (**d**) 10^10^ CFU/day CCFM683 groups. The edge colors indicate positive (red) or negative (green) correlations, which depend on the Pearson’s correlation coefficient. The node size represents the degree.

Moreover, an RMT-based network analysis was used to evaluate each OTUs topological role in microbial networks. The negatively correlated edges in 10^9^ CFU/day (26.02%) and 10^10^ CFU/day (47.10%) CCFM683 groups were much more than those in the control (10.00%) and IMQ (16.18%) group (Figure 11a–d). Moreover, a greater number of connectors in 10^9^ CFU/day (12.90%) and 10^10^ CFU/day (13.64%) CCFM683 groups were discovered compared with those in the control (5.17%) and IMQ (2.70%) groups (Figure S3). The degree was calculated to distinguish the core microbes (Figure 11a–d). *GCA-900066575* (OTU59) and *Lachnospiraceae* (OTU17) were the core microbes in the IMQ group. GCA-900066575 was positively correlated with *Ruminiclostridium 9* (OTU69) and *Ruminiclostridium* 6 (OTU86), and *Lachnospiraceae* was positively correlated with *Negativibacillus* (OTU68) and *Ruminiclostridium 6* (OTU86). However, the *Eubacterium ventriosum group* (OTU70), *Ruminococcaceae UCG-014* (OTU6), and *Rikenellaceae* (OTU32) were the core microbes in control mice, which were different from those in the IMQ group. In mice treated with 10^9^ CFU/day CCFM683, *Bacteroides* (OTU10) was the core microbe and was positively correlated with *Alloprevotella* (OTU11) and *Ruminococcaceae* UCG-010 (OTU74). Therefore, 10^9^ and 10^10^ CFU/day CCFM683 altered the core microbes and rebalanced their interactions.

**Figure 12:**
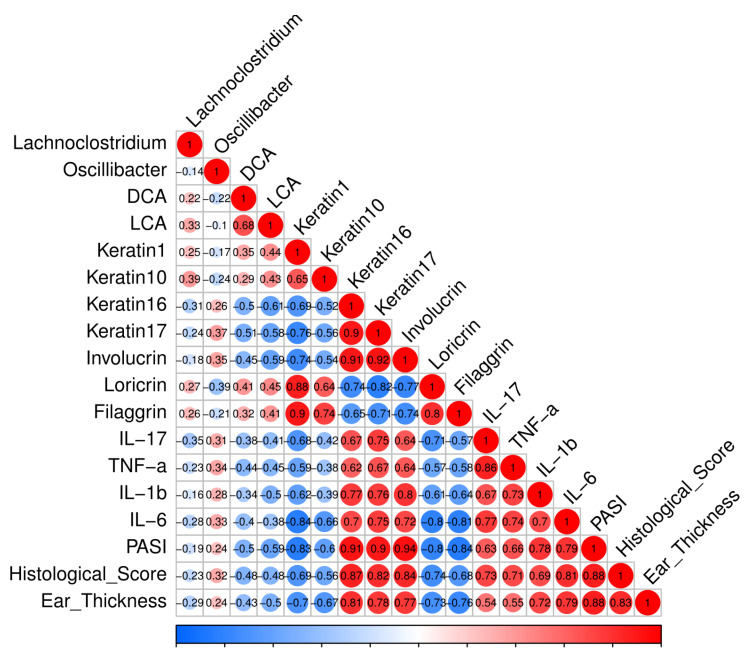
Correlation analysis with R-value of the altered genera, colonic bile acids, psoriasis indices, epidermal structural proteins, and cutaneous cytokines.

Correlations between altered microbes, colonic bile acid concentrations, and psoriasis markers were revealed using Pearson analyses (Figure 12). The concentrations of DCA and LCA were significantly negatively correlated with the cumulative score, histological score, and ear thickness, which were the crucial indicators for psoriasis. This implies that the promotion of DCA and LCA production may be a factor in ameliorating psoriasis in CCFM683-treated mice. *Lachnoclostridium* was positively correlated with keratin 10, indicating its suppressive effect on psoriasis. *Oscillibacter* was positively correlated with keratin 17 and involucrin and negatively correlated with loricrin, indicating that it was contributing to psoriasis development. It was noteworthy that *Lachnoclostridium*, which has been reported to produce DCA in mice, was positively but not significantly correlated with DCA and LCA. Therefore, gut microorganisms might participate in the production of colonic bile acids to help ameliorate psoriasis.

## 4. Discussion

Probiotics have shown regulatory effects in various diseases and these effects can be influenced by the gavage doses of the strain [18,24,25]. However, it remains unclear whether there is a dose–response effect of probiotics in relieving psoriasis. In the current study, the effects of *B. breve* CCFM683 at five doses on ameliorating psoriasis were compared. Treatment with 10^9^ and 10^10^ CFU/day CCFM683 ameliorated psoriasis symptoms (cumulative score and ear thickening) and protected the cutaneous epidermal barrier; however, none of these effects were elicited by 10^6^, 10^7^, or 10^8^ CFU/day CCFM683 treatments. Furthermore, lower PCNA levels (a marker for cell proliferation) were found in 10^9^ and 10^10^ CFU/day CCFM683-treated mice. Thus, there were dose–response effects of *B. breve* CCFM683 on relieving psoriasis symptoms.

Keratinocyte hyperproliferation and parakeratosis, characterized by different kinds of keratins, are the key pathological features of psoriasis [26]. Specifically, keratin 1 and keratin 10 are the structural proteins of the epidermis and are markers for differentiation, whereas keratin 16 and keratin 17 are the hallmarks of proliferation [27]. As previously reported, the supernatants of *B. animalis* CCFM1148 and *L. paracasei* CCFM1147 showed inhibitory effects on the proliferation of HaCaT cells, whereas *B. longum* NCC3001 and *B. longum* NCC2705 extracts increased keratin 1 and keratin 10 in NHEKs [3,28]. However, the regulatory effects of living probiotics on keratins in vivo and their adequate doses have rarely been reported. In the current study, only 10^9^ and 10^10^ CFU/day CCFM683 treatments increased the mRNA levels of keratin 1 and keratin 10 and decreased keratin 16 and keratin 17 levels, while other doses did not elicit these effects. Thus, 10^9^ and 10^10^ CFU/day CCFM683 treatments were able to suppress hyperproliferation and promote normal differentiation in keratinocytes in vivo.

The epidermis is a crucial barrier against environmental pathogens and is damaged during the pathogenesis of psoriasis [29]. Filaggrin and loricrin, which participate in the formation of epidermal cornified envelopes, are decreased in psoriasis, whereas involucrin, another envelope precursor, is overexpressed in psoriasis lesions [30]. In the current study, 10^9^ and 10^10^ CFU/day CCFM683 treatments elicited higher mRNA levels of filaggrin and loricrin, and lower involucrin levels compared with the IMQ treatment group. Similar results were found in *L. plantarum* APsulloc 331261 (1 × 10^8^ CFU/day), which has been shown to improve the integrity and permeability of the epidermal barrier by promoting filaggrin and loricrin expressions [31]. These results were consistent with those in psoriasis patients, who exhibited increased filaggrin and loricrin levels [32,33]. However, 10^6^, 10^7^, and 10^8^ CFU/day CCFM683 treatments did not significantly influence the expression of filaggrin, loricrin, or involucrin compared with IMQ treatment. Therefore, *B. breve* CCFM683 regulates epidermal structural proteins in a dose-dependent manner.

Oral administration of different doses of CCFM683 resulted in different bile acid levels in the colon and the DCA and LCA concentrations were positively correlated with psoriasis alleviation. As previously reported, oral administration of DCA and LCA diminishes the expression of IL-17, thus ameliorating psoriasis in mice [34]. Additionally, interestingly, *Leuconostoc mesenteroides* NTM048 at doses of 10^10^ and 10^11^ CFU/day, which was close to the effective dosage of CCFM683, was able to increase the DCA concentration in serum and relieve psoriasis [7]. Therefore, promoting the production of bile acid may be one of the mechanisms by which *B. breve* CCFM683 relieves psoriasis. Moreover, whether the difference amounts of bile acids were caused by CCFM683 itself, by the changed gut microbiota, or both remains to be further explored.

The farnesoid X receptor is a nuclear receptor that can be activated by bile acids and affects the immune processes in the intestine, liver, and other organs [35]. NF-κB is critically required in keratinocytes and lymphocytes in psoriasis lesions and activates the transcription of downstream genes for chemokines and receptors participating in the immune responses in psoriasis [36]. It has been reported that FXR activation inhibits NF-κB nuclear translocation in various diseases [37,38]. Here, we confirmed that 10^9^ and 10^10^ CFU/day CCFM683 up-regulated the FXR expression and diminished the crucial molecules of the NF-κB pathway, whereas 10^6^, 10^7^, and 10^8^ CFU/day CCFM683 treatments did not. Thus, the regulation of the FXR/NF-κB pathway might be a key factor in the amelioration of psoriasis by CCFM683, and the regulatory effects also followed a dose-dependent manner.

Cutaneous immune response disorder is an important characteristic of psoriasis [39]. IL-1β and IL-6 have been confirmed to promote the differentiation of CD4+T cells into Th17 cells, in which IL-17 is produced, and higher IL-17 levels recognized by keratinocytes leads to severe hyperkeratosis and inflammatory infiltration [40]. Chemokines, including CCL3 and CCL5, participate in the pathogenesis of psoriasis by recruiting and activating T cells, macrophages, and neutrophils [41]. In mice with psoriasis, *L. pentosus* GMNL-77 (2 × 10^9^ CFU/day) down-regulated the mRNA level of IL-6, which improved psoriasis symptoms [5]. Moreover, the production of IL-17 was decreased in psoriasis by 5 × 10^9^ CFU/day *B. breve* CCFM1078 [6]. In the present study, 10^9^ and 10^10^ CFU/day CCFM683 significantly decreased the cutaneous concentrations of IL-17, IL-1β, IL-6, and TNF-α, as well as suppressing the expressions of CCL3, CCL5, CCL8, and G-CSF in the skin. However, there were no such effects in 10^6^, 10^7^, and 10^8^ CFU/day CCFM683 treatments. Thus, regulating inflammatory cytokines and chemokines was an important mechanism for *B. breve* CCFM683 in relieving psoriasis, the effects of which were influenced by the gavage dosage.

The gut microbiota has been reported to play a crucial role in the development of skin diseases [42]. The diversity of gut microbiota in psoriasis patients is much lower than that in healthy individuals [43]. After 10^9^ CFU/day CCFM683 or 10^10^ CFU/day CCFM683 treatment, the gut microbiota diversity recovered and the microbial profiles were changed. The relative abundances of Firmicutes were elevated and Bacteroidetes were diminished in both 10^9^ and 10^10^ CFU/day CCFM683 groups. It has been reported that the Firmicutes/Bacteroidetes ratio is significantly higher in psoriasis patients compared to healthy individuals and positively related to PASI [43], which was consistent with the current results. At the genus level, both 10^9^ and 10^10^ CFU/day CCFM683 significantly increased the relative abundance of *Lachnoclostridium*. *Lachnoclostridium* has been reported to express the enzyme 7α-hydroxylase that drives the conversion of cholic acid to DCA, thus having a potential role in ameliorating psoriasis [44]. Here, although not significantly, the relative proportion of *Lachnoclostridium* was positively correlated with DCA and LCA, which implied that certain bacteria in the gut microbiota might produce secondary bile acids, thus helping to improve psoriasis. Meanwhile, the proportion of *Oscillibacter* was significantly reduced in 10^9^ and 10^10^ CFU/day CCFM683 groups. *Oscillibacter* produces TLR2-ligands and induces Mmp2 expression via the MYD88-ATF3 pathway, resulting in mitochondrial damage and inflammation in cells [45]. Consistently, the relative abundance of *Oscillibacter* was positively correlated with cutaneous cytokines, implying that it may aggravate the inflammatory responses in psoriasis mice. Therefore, 10^9^ CFU/day and 10^10^ CFU/day CCFM683 treatments increased DCA-producing *Lachnoclostridium* levels and decreased harmful *Oscillibacter* levels, which might be an important factor in psoriasis alleviation.

Moreover, the gut microbiota in psoriasis patients has been reported to be disorganized and unbalanced [46]. In the current study, an unbalanced network was found in IMQ-treated mice, the core microbes of which were *GCA-900066575* and *A2*. *GCA-900066575* was positively correlated with *Ruminiclostridium 9* and *Ruminiclostridium 6*, which are positively correlated with inflammatory indicators in various diseases [47,48], and *A2* was positively correlated with *Negativibacillus*, a biomarker for colonic inflammation [49]. However, the unbalanced microbial profiles were recovered by 10^9^ and 10^10^ CFU/day CCFM683 treatments. In the 10^9^ CFU/day CCFM683 group, *Bacteroides* represented the core microbe and was positively correlated with *Alloprevotella* and *Ruminococcaceae UCG-010*. *Alloprevotella* was reportedly decreased in colitis and is negatively correlated with proinflammatory cytokines [50]. *Ruminococcaceae UCG-010* was reported to promote the production of secondary bile acids, especially DCA, which have a beneficial effect on host health [51]. Co-occurrence networks had more negative correlations in the 10^9^ CFU/day or 10^10^ CFU/day CCFM683 group compared with those of the IMQ group, which can be interpreted as increased inter-species competition against “psoriatic microbes” after CCFM683 treatment. Thus, 10^9^ and 10^10^ CFU/day CCFM683 treatments improved the unbalanced microbiota and regulated the core microbiota, which was another factor in improving psoriasis.

## 5. Conclusions

In the current study, *B. breve* CCFM683 at the doses of 10^9^ and 10^10^ CFU/day significantly ameliorated psoriasis in mice, and the protective effects were significantly related to the gavage dose. According to the dose–effect curves, a gavage dose of more than 10^8.42^ CFU/day was required for CCFM683 to relieve psoriasis in mice. The multiple mechanisms of action include diminishing inflammatory cytokines, regulating the proliferation and differentiation of keratinocytes, protecting the epidermal barrier via increasing loricrin and filaggrin, promoting the production of bile acids, regulating the diversity of the gut microbiota, increasing beneficial bacteria and diminishing harmful bacteria, and altering core microbes and their interactions. These results may be helpful for the understanding of the mechanism of psoriasis amelioration by CCFM683, thus contributing to clinical trials and probiotic product development. However, the conversion of probiotic doses between mice and humans is not clear at present; therefore, clinical trials should be conducted to investigate the efficacy of CCFM683 in psoriasis patients in the future.

## Figures and Tables

**Table 1:** Animal Experiment Design.

Group	Daily Gavage Treatment (0.2 mL)	15–20 Days
Control	Saline (0.85%)	Treated with vaseline
IMQ	Saline (0.85%)	Treated with IMQ
MTX	2 mg/mL methotrexate	Treated with IMQ
10^6^ CFU/day CCFM683	5 × 10^6^ CFU/mL CCFM683	Treated with IMQ
10^7^ CFU/day CCFM683	5 × 10^7^ CFU/mL CCFM683	Treated with IMQ
10^8^ CFU/day CCFM683	5 × 10^8^ CFU/mL CCFM683	Treated with IMQ
10^9^ CFU/day CCFM683	5 × 10^9^ CFU/mL CCFM683	Treated with IMQ
10^10^ CFU/day CCFM683	5 × 10^10^ CFU/mL CCFM683	Treated with IMQ

**Table 2:** Primers Used in the Real-Time PCR.

Gene	Forward Primer (5′–3′)	Reverse Primer (5′–3′)
*k1*	TGGGAGATTTTCAGGAGGAGG	GCCACACTCTTGGAGATGCTC
*k10*	CTGGCGATGTGAACGTGGAA	GTCCCTGAACAGTGCGTCTC
*k16*	GGTGGCCTCTAACAGTGATCT	TGCATACAGTATCTGCCTTTGG
*k17*	ACCATCCGCCAGTTTACCTC	CTACCCAGGCCACTAGCTGA
*Lor*	GCGGATCGTCCCAACAGTATC	TGAGAGGAGTAATAGCCCCCT
*Ivl*	ATGTCCCATCAACACACACTG	TGGAGTTGGTTGCTTTGCTTG
*Flg*	ATGTCCGCTCTCCTGGAAAG	TGGATTCTTCAAGACTGCCTGTA
*G-CSF*	ATGGCTCAACTTTCTGCCCAG	CTGACAGTGACCAGGGGAAC
*CCL3*	TTCTCTGTACCATGACACTCTGC	CGTGGAATCTTCCGGCTGTAG
*CCL5*	GCTGCTTTGCCTACCTCTCC	TCGAGTGACAAACACGACTGC
*CCL8*	TCTACGCAGTGCTTCTTTGCC	AAGGGGGATCTTCAGCTTTAGTA
*β-actin*	GGCTGTATTCCCCTCCATCG	CCAGTTGGTAACAATGCCATGT

## Data Availability

Data sharing not applicable.

## Supplementary Materials

The following supporting information can be downloaded at: https://www.mdpi.com/article/10.3390/nu15081952/s1, Figure S1: Effect of CCFM683 treatment on (a) UDCA, (b) TUDCA, (c) HDCA, (d) β-MCA, (e) TCA, and (f) CA in the colon. n = 8 mice per group; Figure S2: Quantitative relationships between the colonic LCA concentration and (a) ear thickness, (b) PASI and (c) histological score; Figure S3: Zi-Pi plot showing the distribution of genera based on their topological roles in networks. Each symbol represented an OTU in the bacterial network. The threshold values of Zi and Pi for categorizing genus were 2.5 and 0.62, respectively.
