# Supplementary material for: Dose–Response Efficacy and Mechanisms of Orally Administered Bifidobacterium breve CCFM683 on IMQ-Induced Psoriasis in Mice
Source: Nutrients. 2023 Apr 18;15(8):1952. doi: 10.3390/nu15081952 (PMC10143451; doi:10.3390/nu15081952)
Supplement: Supplementary file 1 [file nutrients-15-01952-s001.zip › nutrients-2290820-supplementary.pdf]

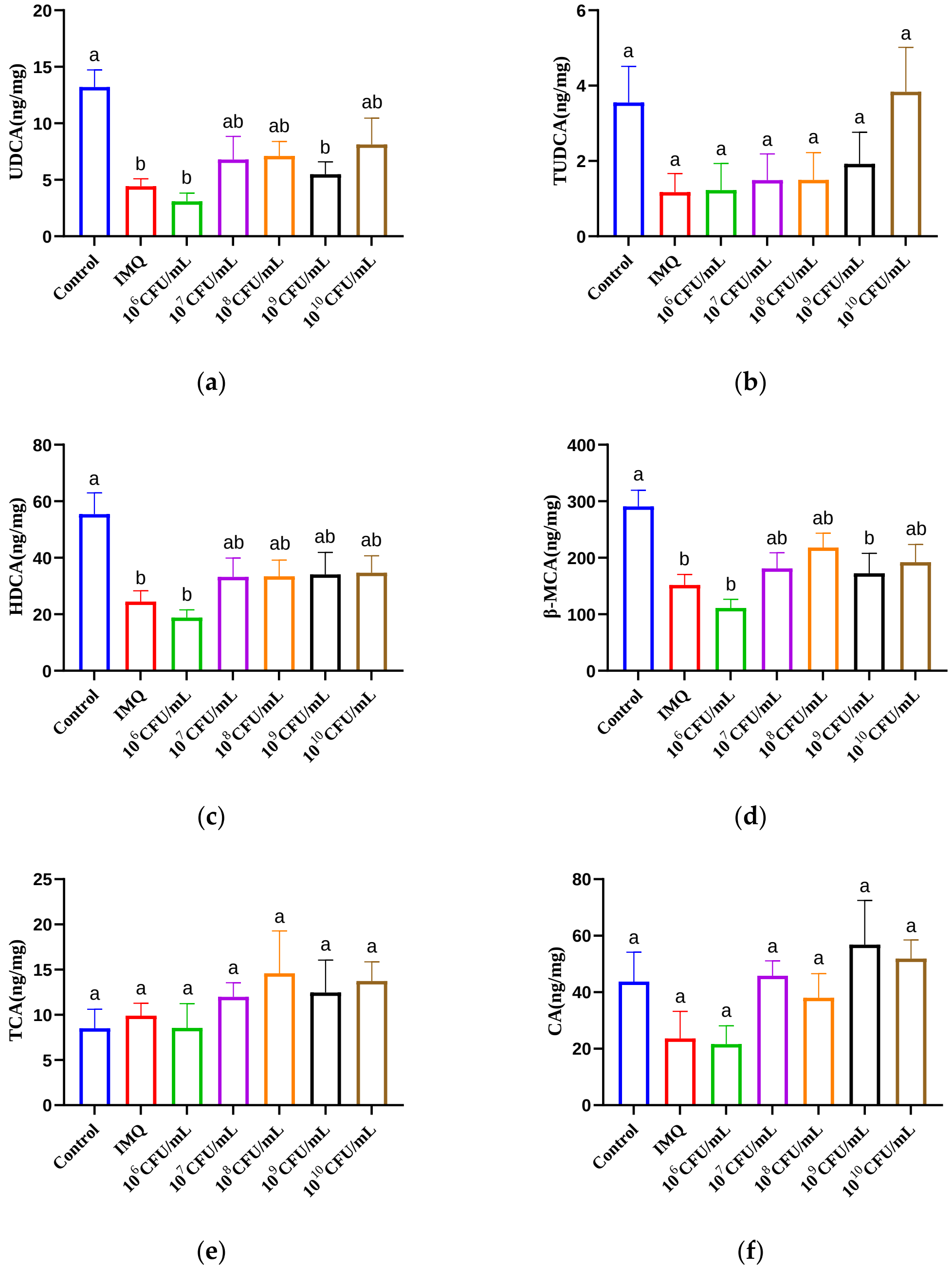

Figure S1. Effect of CCFM683 treatment on (a)UDCA, (b)TUDCA, (c)HDCA, (d)β-MCA, (e)TCA, and (f)CA in the colon. n = 8 mice per group.

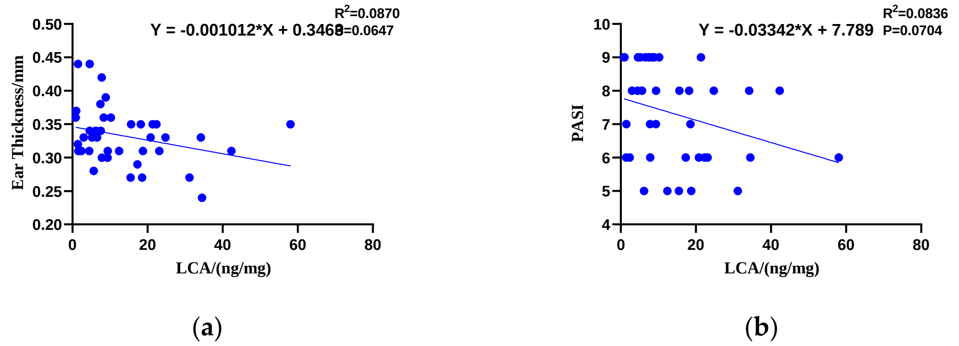

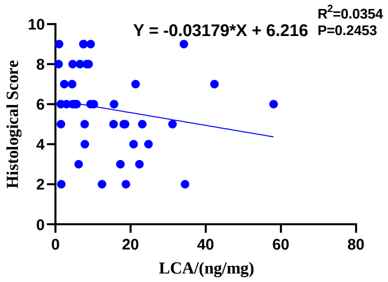

(c)

Figure S2. Quantitative relationships between the colonic LCA concentration and (a) ear thickness, (b) PASI and (c) histological score.

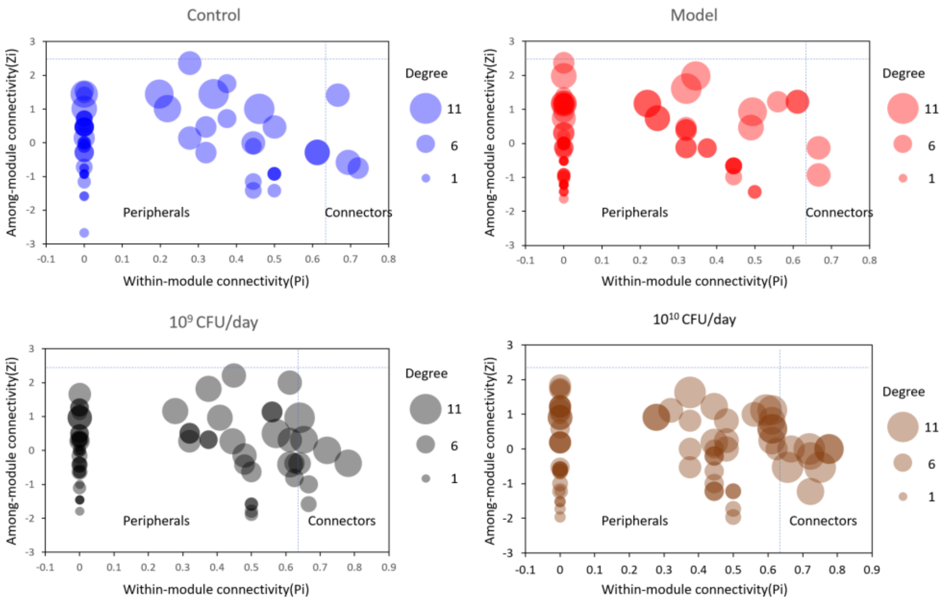

Figure S3. Zi-Pi plot showing the distribution of genera based on their topological roles in networks. Each symbol represented an OTU in the bacterial network. The threshold values of Zi and Pi for categorizing genus were 2.5 and 0.62, respectively.
